# Contribution of Underlying Connective Tissue Cells to Taste Buds in Mouse Tongue and Soft Palate

**DOI:** 10.1371/journal.pone.0146475

**Published:** 2016-01-07

**Authors:** Kristin Boggs, Nandakumar Venkatesan, Ingmar Mederacke, Yoshihiro Komatsu, Steve Stice, Robert F. Schwabe, Charlotte M. Mistretta, Yuji Mishina, Hong-Xiang Liu

**Affiliations:** 1 Regenerative Bioscience Center, Department of Animal and Dairy Science, College of Agricultural and Environmental Sciences, University of Georgia, Athens, GA, United States of America; 2 Department of Medicine, College of Physicians and Surgeons, Columbia University, New York, NY, United States of America; 3 Department of Pediatrics, Medical School, The University of Texas Health Science Center at Houston, Houston, TX, United States of America; 4 Department of Biologic and Materials Sciences, School of Dentistry, University of Michigan, Ann Arbor, MI, United States of America; University Of Melbourne, AUSTRALIA

## Abstract

Taste buds, the sensory organs for taste, have been described as arising solely from the surrounding epithelium, which is in distinction from other sensory receptors that are known to originate from neural precursors, i.e., neural ectoderm that includes neural crest (NC). Our previous study suggested a potential contribution of NC derived cells to early immature fungiform taste buds in late embryonic (E18.5) and young postnatal (P1-10) mice. In the present study we demonstrated the contribution of the underlying connective tissue (CT) to *mature* taste buds in mouse tongue and soft palate. Three independent mouse models were used for fate mapping of NC and NC derived connective tissue cells: (1) *P0-Cre/R26-tdTomato (RFP)* to label NC, NC derived Schwann cells and derivatives; (2) *Dermo1-Cre/RFP* to label mesenchymal cells and derivatives; and (3) *Vimentin-CreER/mGFP* to label Vimentin-expressing CT cells and derivatives upon tamoxifen treatment. Both *P0-Cre/RFP* and *Dermo1-Cre/RFP* labeled cells were abundant in mature taste buds in lingual taste papillae and soft palate, but not in the surrounding epithelial cells. Concurrently, labeled cells were extensively distributed in the underlying CT. RFP signals were seen in the majority of taste buds and all three types (I, II, III) of differentiated taste bud cells, with the neuronal-like type III cells labeled at a greater proportion. Further, *Vimentin-CreER* labeled cells were found in the taste buds of 3-month-old mice whereas Vimentin immunoreactivity was only seen in the CT. Taken together, our data demonstrate a previously unrecognized origin of taste bud cells from the underlying CT, a conceptually new finding in our knowledge of taste bud cell derivation, i.e., from both the surrounding epithelium and the underlying CT that is primarily derived from NC.

## Introduction

Sensory receptors, as part of the peripheral nervous system, are known to arise from neurogenic ectoderm that includes the neural tube, neural crest (NC) or ectodermal placodes [1, 2]. Thus, receptor organs, in general, have neural progenitors that migrate and differentiate locally to specific receptors. In contrast, taste bud cells have been described on the basis of anatomical studies [3, 4] and transgenic phenotype analyses [5, 6] as arising solely from the local epithelium [7]. However, the heterogeneity of structural (types I, II, III, IV) [8–10] and functional (epithelial-, neuronal-, and glial-like) [11–14] cell features indicate distinct lineages of taste bud cells [15].

The use of a tissue- or inducible tissue-specific *Cre/loxP* recombinase system has significantly advanced our knowledge pertaining to taste bud precursor/progenitor cell constitutions and how specific tissues/cell populations regulate the formation and maintenance of taste organs. Cell fate mapping using an inducible *Cre/loxP* system driven by a sonic hedgehog promoter (*Shh-CreER*) demonstrated that Shh-expressing embryonic taste papilla placodes [16] and basal cells of taste buds [17] are precursors of differentiated taste cells. With a *Gli1-CreER* mouse, populations of hedgehog-responding and *Gli1* labeled progeny cells in basal epithelium and connective tissue core of the fungiform papilla were shown to contribute to maintenance of fungiform papillae and taste buds [18]. Moreover, use of an *Lgr5-CreER* mouse model provided evidence that Lgr5-expressing cells in the basal region of taste buds are precursors of taste bud cells [19]. Furthermore, *Wnt1-Cre*, a well characterized and widely used mouse model for labeling NC cells and their derivatives [20], has been a useful tool in demonstrating the contribution of NC to tongue mesenchyme and the important roles of NC derived cells in tongue myogenesis and morphogenesis [21].

In a study to demonstrate that taste bud cells are derived from the local surrounding epithelium, an inducible *Cre* driven by the promoter of K14 (*K14-CreER*) was used and Cre-labeled cells were analyzed in postnatal mice [5]. K14 is a basal epithelial cell marker, and *K14-CreER* labeled a population, but not all, of taste bud cells. Even after 1-month of pulse chase period that goes beyond the turnover cycle of all taste bud cells [22], only a subset of taste bud cells were labeled. Moreover, it was shown in the report that absence of taste bud labeling was frequently observed concurrently with the labeling of surrounding epithelial cells. The data strongly suggest other source(s) of progenitor/stem cells for taste bud formation and renewal. In mouse tongue and soft palate, taste buds reside in the epithelium that overlys a layer of loose connective tissue (lamina propria). Therefore, taste buds are structurally surrounded by both local epithelium and underlying connective tissue (CT) which is potentially another precursor source.

Our recent findings using DNA recombination-based cell lineage tracing studies in *P0-Cre* and *Wnt1-Cre* mice [23] suggest a potential NC contribution to early immature taste buds at embryonic day 18.5 (E18.5) and postnatal day 1–10 (P 1–10). However, the difference was profound in the proportions of *P0-Cre* (abundant) and *Wnt1-Cre* (sparse) labeled cells in early taste buds. Further evidence is needed to confirm this significant finding and questions remain about (1) whether *mature* taste bud cells are derived from the underlying mesenchymal CT; (2) whether underlying CT contributes to specific taste cell type(s); and (3) whether there are stem/progenitor cells in the underlying CT that continuously contribute to the renewal of mature taste buds.

To address these questions, we used three independent mouse lines for the present study: *P0-Cre* which labels a population of NC cells, Schwann cells and a small portion of cortex that are derived from the periventricular cells [24–26]; *Dermo1-Cre* (*Twist2-Cre*) [27], in which *Cre* recombinase is driven by the endogenous promoter of *Dermo1* that is expressed in the mesenchymal cells [28]; and *Vimentin-CreER* with an inducible *Cre* driven by the endogenous promotor of *Vimentin* [29], that is exclusively expressed in the mesenchyme and mesenchymal CT in embryonic [30] and adult mouse tongue. We found labeled cells abundantly distributed in mature taste buds concurrently with the distribution in underlying CT, but not in the surrounding epithelium. Our data support a new concept, i.e., taste bud cells are derived from both the underlying CT and the surrounding epithelium. This new finding brings a better understanding of progenitor sources of taste bud formation and renewal.

## Materials and Methods

### Animals

The use of animals was approved by The University of Georgia Institutional Animal Care and Use Committee and was in compliance with the National Institutes of Health Guidelines for care and use of animals in research.

The hemizygous *P0-Cre* mouse line [26], C57BL6J-*Tg(P0-Cre)94Img* (ID148), was provided by CARD, Kumamoto, Japan. *Dermo1-Cre* (B6.129X1-*Twist2*^*tm1*.*1(cre)Dor*^*/J*) [27] was purchased from Jackson Laboratory (Stock#008712). *Vimentin-CreER* was generated by Dr. Schwabe [29]. *P0-Cre* and *Dermo1-Cre* mice were bred with homozygous *R26-tdTomato* (*RFP*) reporter mice (B6.Cg-*Gt(ROSA)26Sor*^*tm14(CAG-tdTomato)Hze*^*/J*, Jackson Lab, Stock #007914). *Vimentin-CreER* mice were bred with cell membrane-targeted, two-color fluorescent Cre reporter allele (Rosa^mTom/mGFP^, *Gt(ROSA)26Sor*^*tm4(ACTB-tdTomato*,*-EGFP)Luo*^/J, Jackson Lab, Stock#007676).

PCR genotyping was conducted to detect *Cre*, *RFP* and *GFP*. In brief, DNA from the tail tissue was extracted with 50 mM sodium hydroxide at 98°C for 30 min and neutralized with Tris-HCl. PCR amplification was carried out with diluted DNA (1:20) under the conditions of denaturation at 94°C for 5 min followed by annealing at 58–69°C for 30 s and extension at 72°C for 30 s; this cycle was repeated 40 times. PCR products were visualized in 2% agarose gel electrophoresis.

Male and female mice were grouped together because no apparent difference was observed in the distribution of labeled cells between two genders. *Cre* negative littermates served as controls.

### Tissue collection

Postnatal *P0-Cre/RFP* mice at different stages were used for tissue collections, i.e., 2 weeks when mature taste buds are developing (maturing stage); 4 weeks when taste buds are mature (mature stage); 8 weeks young adult and 16 weeks mature adult when taste bud cells undergo continuous turnover for the maintenance of proper function of taste (turnover stage). Adult (3–4 months) *Dermo1-Cre* mice were used. *Vim-CreER* activity was induced by 4 intraperitoneal injections of tamoxifen (0.08 mg/g body weight dissolved in corn oil, administered every 3–4 days) to induce cell membrane-localized green fluorescence (mGFP) in *Vimentin* expressing cells and derived cells in adult mice. Tissues from 3-month-old mice were collected 2 days after the last tamoxifen injection, i.e., 12 days after the first tamoxifen injection.

Mice were euthanized with CO_2_ followed by cervical dislocation. Following transcardial perfusion with warm 0.1 M phosphate buffered saline (PBS) solution, warm 2% paraformaldehyde (PFA) in PBS (pH 7.4) and cold 2% PFA, the whole tongue and soft palate were collected and post-fixed in 2% PFA in PBS at 4°C for 3–5 hr, then transferred to 30% sucrose in 0.1 M PBS at 4°C for approximately 48 hr. For the double immunolabeling of Vimentin and Ki67, fresh tissues were collected immediately after euthanization.

The whole tongue was dissected into the following pieces: two halves (from lateral edge to the midline of median furrow) of the anterior 2/3 oral tongue containing fungiform papillae and taste buds, two lateral edges of the posterior oral tongue where foliate papillae and taste buds are located, and tissue containing the single circumvallate papilla in the mid-line of the border between the oral and pharyngeal tongue. The tissues were embedded in O.C.T. compound (Tissue Tek) and frozen for cryostat sectioning at different orientations: sagittal for fungiform and foliate taste bud tissues, and transverse for circumvallate tissues. Soft palate tissues were oriented for sagittal sections. Serial (fungiform and soft palate) and neighboring (circumvallate and foliate) sections were cut at 5-μm thickness, mounted onto charged slides and processed further for immunohistochemistry.

### Immunohistochemistry

Primary antibodies used were: α-Gustducin (1:1000, sc-395, Santa Cruz Biotechnology, TX), GFP (1:500, Life Technologies, NY), Keratin 8 (Krt8) (1:1000, TROMA-I, Developmental Studies Hybrydoma Bank, IA), Ki67 (1:200, ab16667, ABCAM, MA), NTPDase II (1:1000, Centre de recherché du CHUL Rhumatologie-Immunologie, Québec, Canada), SNAP-25 (1:5000, S9684, Sigma-Aldrich, MO), and Vimentin (1:1000, AB5733, EMD Millipore, MA). Slides without primary antibody treatment were used as controls.

In brief, fungiform, foliate, circumvallate and soft palate tissue sections were air dried for 1 hr at room temperature and rehydrated in 0.1 M PBS. Blocking of nonspecific staining was carried out by incubation with 10% normal donkey serum in PBS containing 0.3% Triton X-100 (Sigma, St. Louis, MO) for 30 min. Then the sections were incubated with primary antibody in the carrier solution (1% normal donkey serum, 0.3% Triton X-100 in PBS) overnight at 4°C. Following rinsing in 0.1 M PBS, sections were incubated in Alexa Fluor^®^ 488 (for GFP) or 647 (for all the other markers)-labeled secondary antibody (1:500, Invitrogen, Eugene, OR) for 1 hr at room temperature. Sections were rinsed with PBS and counterstained with DAPI (200 ng/ml in PBS) for 10 min. After thorough rinsing in PBS, the slides were air dried and cover slipped with Prolong^®^ Gold antifade mounting medium (Invitrogen, Eugene, OR). The sections were analyzed under light microscope (EVOS FL, Life Technologies). Co-localization of RFP and pan- or type-specific taste cell markers (Krt8, NTPDaseII, αGustducin, SNAP25) was confirmed and photographed using a laser scanning confocal microscope (Zeiss LSM 710 and 510).

### Quantification of *P0-Cre* labeled taste bud cells

Quantitative analyses were made in *P0-Cre/RFP* tissues to calculate: (1) the proportion of RFP^+^ taste bud cells in fungiform papillae at different stages (2, 4, 8 and 16 weeks); (2) the proportion of RFP^+^ cells in foliate and circumvallate taste buds at week 8; and (3) the proportion of RFP^+^ cells in different taste cell types in fungiform, foliate and circumvallate taste buds at week 8. Taste bud cells labeled by bright red fluorescence protein (RFP^+^), or/and Krt8, α-Gustducin, SNAP25 immunoproducts were counted. Only cells with a clear nucleus labeled by DAPI were included.

Fungiform taste papillae have a patterned array in the anterior oral tongue, and each papilla contains a single taste bud that may be tracked in serial sections. Serial sagittal sections at 5-μm thickness were collected from the anterior half of the oral tongue (from lateral edge to the midline of median furrow) at 2, 4, 8 and 16 weeks (n = 3 mice per stage). Sections were immunoreacted with a pan taste cell marker, Krt8. Individual taste buds were tracked in serial sections and taste buds with all sections available for counting were included for further analysis. An EVOS FL multichannel fluorescence microscope and software were used for the quantification by one investigator. Krt8 signals were used for marking the boundary of differentiated taste bud cells on each section. The total number of DAPI stained nuclei in a Krt8^+^ taste bud in all sections was determined for each bud, and described as the “cell profile” number of a taste bud. In the same manner, RFP^+^ taste bud cells, each with a clear nucleus, were also quantified in all sections and totaled for each taste bud. The percentage of *P0-Cre* labeled RFP^+^ taste bud cell profiles was obtained by dividing the total RFP^+^Krt8^+^ taste bud cell profile number by the total Krt8^+^ taste bud cell profile number per taste bud.

Taste buds in the foliate and circumvallate papillae are numerous and close to each other, making individual taste buds difficult to track. Therefore, the overall numbers of total RFP^+^Krt8^+^ taste bud cell profiles and total Krt8^+^ taste bud cell profiles in all taste buds on a section were quantified. The proportion of RFP^+^ taste bud cells was represented by the percentage of RFP^+^Krt8^+^ relative to Krt8^+^ taste cell profile numbers.

For the proportion of RFP^+^ taste bud cell types, the RFP^+^ type II (α-Gustducin^+^) and RFP^+^ type III (SNAP25^+^) taste cells were quantified, and the percentage was calculated relative to total RFP^+^ taste bud cells, or total α-Gustducin^+^ type II or SNAP25^+^ type III cells. Type I cells (NTPDaseII^+^) comprised the majority of taste bud cells, and individual NTPDaseII^−^ cells were difficult to identify. Therefore, the percentage of RFP^+^ type I cells was extrapolated based on the quantification data for type II and III cells.

### Statistical Analysis

**Fig 1:**
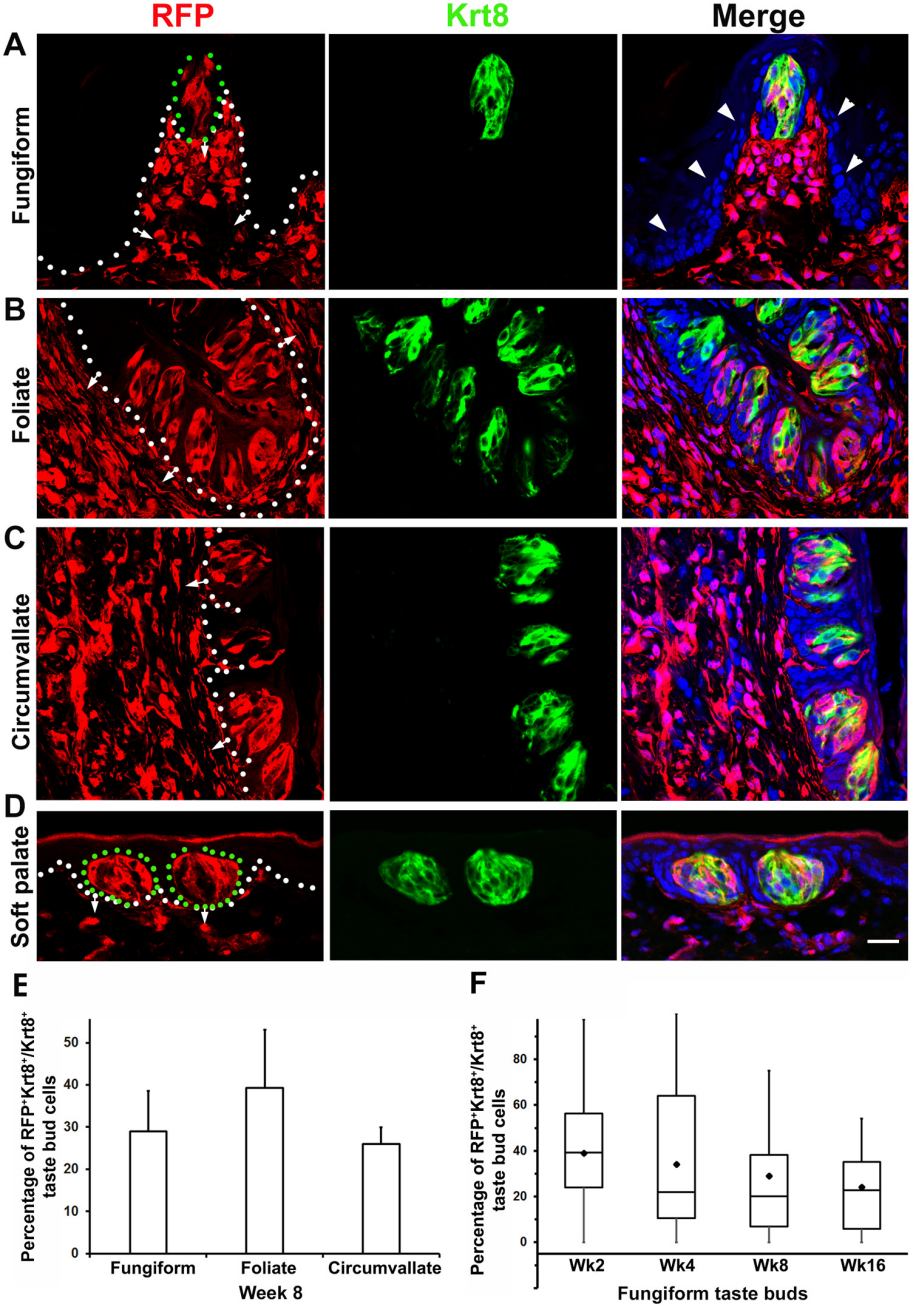
A-D: Single-plane laser-scanning confocal photomicrographs illustrate the distribution of RFP^+^ cells in mature taste buds in young adult (8 week) *P0-Cre/RFP* mice. Taste bud cells in lingual fungiform (A), foliate (B), circumvallate (C) papillae and soft palate (D) were labeled by immunoreactivity of a pan-taste cell marker Keratin 8 (Krt8, green). Tissue sections were counterstained with DAPI (blue) to stain the nuclei of all cells. White dotted lines demarcate the epithelium from underlying connective tissue with short arrows pointing to connective tissue. Green dots in A and D bracket taste buds. *P0-Cre* driven RFP^+^ cells were abundantly distributed in taste buds and underlying lamina propria of the tongue and soft palate. No RFP^+^ cells were seen in the surrounding epithelium (arrowheads) of taste buds. Scale bars: 20 μm for all images. **E**: Histogram shows the average (x̄±SD, n = 3) of RFP^+^Krt8^+^ as a proportion of total Krt8^+^ taste bud cell profiles in fungiform, foliate and circumvallate papillae in 8-week-old mice. **F**: Data from 3 mice for each stage (2, 4, 8 and 16 week) are represented as box plot of median±percentile. The diamond within each box represents the average (n = 3) of RFP^+^Krt8^+^ double labeled versus total Krt8^+^ taste bud cell profiles in fungiform papillae at different stages.

The percentages of the subset of *P0-Cre* labeled RFP^+^ taste bud cells relative to total Krt8^+^ taste bud cells in fungiform papillae are presented as means ± standard derivation ($\bar{X}$ ± SD) and illustrated in Fig 1E and 1F (diamonds within boxes). Also, the percentages of the subset of *P0-Cre* labeled RFP^+^ taste bud cells relative to total Krt8^+^ taste bud cells in fungiform papillae at different stages were plotted as median ± percentile in order to illustrate the distribution of percentages (Fig 1F). One-way analysis of variance (ANOVA) was used to evaluate statistical difference across groups. A *P*-value less than 0.05 is taken as statistical significance.

## Results

### Abundant distribution of *P0-Cre/RFP* labeled cells in taste buds and underlying connective tissue in the tongue and soft palate

#### *P0-Cre/RFP* labeled cells were sustained in *mature* taste buds in young adult mice

To map the fate of *P0-Cre/RFP* labeled cells in *mature* taste buds, tongue and soft palate, tissues were collected in young adult (8-week old) *P0-Cre/RFP* mice. Taste bud cells in all three types of lingual taste papillae, i.e., fungiform, foliate, and circumvallate, and in soft palate were labeled by a pan-taste cell marker, Krt8. No RFP^+^ cells were seen in the epithelium or connective tissues (CT) of tongue or soft palate in the *Cre*-negative littermates (S1 Fig).

In the epithelium of the tongue and soft palate, RFP^+^ cells labeled by *P0-Cre* were frequently seen in mature taste buds in all three types of lingual taste papillae, i.e., fungiform (Fig 1A), foliate (Fig 1B) and circumvallate (Fig 1C), and in the soft palate (Fig 1D). The RFP signals in the structurally recognized taste buds were co-localized with the pan-taste cell marker Krt8. Significantly, RFP^+^ cells were not seen in the lingual and palatal epithelium outside of the taste buds, i.e., neither in the epithelial cells that immediately surround taste buds (Fig 1A, arrowheads) nor in between-papilla lingual or between-bud palatal epithelium (S2 Fig arrowheads).

In the CT of the tongue and soft palate, RFP^+^ cells were extensively distributed, more densely in the core of taste papillae and lamina propria of the tongue and soft palate (Fig 1A–1D, short arrows). In contrast, striated muscle cells, known as mesodermal derivatives, were not RFP^+^ (S2 Fig Fungiform, arrows). Compared to RFP labels in tongue lamina propria, RFP^+^ cells in the palate CT were less dense (Fig 1D).

The abundant distribution of *P0-Cre/RFP* labeled cells in taste buds and underlying CT, in the absence of labeled surrounding epithelial cells suggests that a large population of taste bud cells has the same origin as the underlying CT, which is primarily from cranial neural crest (NC).

#### Proportion and distribution of *P0-Cre/RFP* labeled cells in mature taste buds

To evaluate the contribution of underlying CT-originating cells (potentially NC) to taste buds in a quantitative manner, we examined the proportion of *P0-Cre* labeled taste cell profiles in three types of taste papillae, i.e., fungiform, foliate, and circumvallate. At week 8, the percentages of RFP^+^ taste bud cell profiles versus total Krt8^+^ cell profiles were 29±10% (fungiform), 39±14% (foliate) and 26±4% (circumvallate) (Fig 1E). No statistically significant differences in the proportions of RFP^+^ taste bud cells were found in the three types of taste papillae (F(2,6) = 1.47, *P* = 0.30).

Each of the fungiform taste papillae in mouse tongue contains a single taste bud that may be tracked in serial sections which enables us to examine the proportion of RFP^+^ cells in individual taste buds. Fungiform taste buds were analyzed across a broad range of stages, i.e., taste bud not fully mature at postnatal week 2 (S3A Fig), mature at week 4 (S3B Fig), young adult at week 8 (Fig 1A) and mature adult at week 16 (S3C Fig). Consistent with the observations in young adult (8 weeks) *P0-Cre/RFP* mice (Fig 1A), the distribution of *P0-Cre* labeled RFP^+^ cells in fungiform taste buds was concurrent with extensive labeling in the underlying CT in young pups (week 2–4) and mature adult (week 16) mice (S3A, S3B and S3C Fig). Again, no RFP^+^ cells were seen in the tongue epithelium (arrowheads) outside of the taste buds.

Throughout all stages used (week 2, 4, 8, 16; n = 3 per stage), 88–96% of *fungiform* taste buds contained RFP^+^ cells. However there was considerable variability in the proportion of RFP^+^ taste bud cells among taste buds for each mouse at all stages (Fig 1F, median±percentile plots). At week 2 and 4, 5% of buds were fully labeled, and over 30% of taste buds were more than 50% labeled with RFP^+^ taste bud cells. A small proportion (4%) of taste buds had no RFP^+^ cells at weeks 2–4. In adult mice (8–16 weeks), 83% of examined taste buds had up to half of taste bud cells labeled with RFP; no taste buds were fully labeled, and 12% were not labeled at all. At week 2, 4, 8, 16, RFP^+^ *fungiform* taste bud cell profiles comprised 39±10 (x̄±SD), 34±14, 29±10, and 24±5% of total Krt8^+^ cell profiles respectively (Fig 1F). No statistically significant difference was found among the four stages (F(3,8) = 2.43, *P* = 0.14).

#### Type I, II, III taste cells were labeled by *P0-Cre* in lingual and palatal taste buds

**Fig 2:**
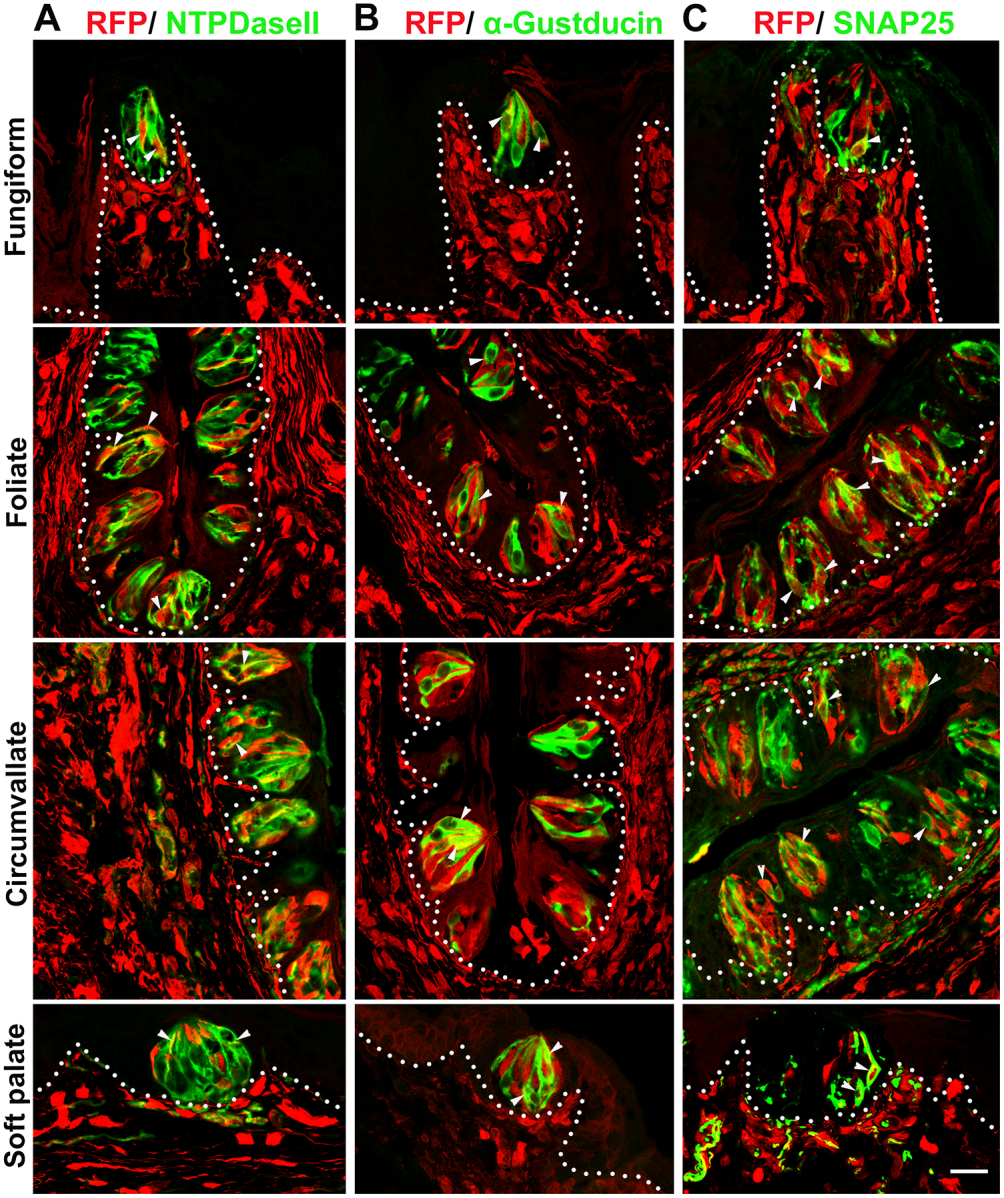
*P0-Cre* labeled type I, II, III taste bud cells. In lingual and palatal taste buds of adult *P0-Cre/RFP* mice, RFP^+^ signals were co-localized with markers for specific taste cell types (white arrowheads), i.e., NTPDaseII for type I cells (A), α-Gustducin for type II cells (B) and SNAP25 for type III cells (C). White dotted lines demarcate the epithelium from underlying connective tissue. Scale bar: 20 μm for all images (single plane laser-scanning confocal).

Taste buds include distinct cell types with proposed functional roles. To determine whether *P0-Cre* labeled cells contribute to specific taste bud cell type(s), we used specific markers to label differentiated taste bud cells, i.e., NTPDaseII for type I, α-Gustducin for type II, and SNAP25 for type III cells, in adult (8–16 week) *P0-Cre/RFP* mice, in which the distribution and proportion of labeled taste bud cells were stable at these stages (Fig 1F). Consistently, labeled RFP^+^ cells were found in taste buds and underlying CT. Co-localization of RFP labels with markers for type I, II and III taste cells was found in all three types of lingual taste papillae and in the soft palate in adult *P0-Cre/RFP* mice (Fig 2, arrowheads).

Type I, II, III cells are within taste buds in different proportions, with type I the most and III the least abundant [31]. Consistently, NTPDaseII labeled the majority of taste bud cells and some nerve fibers in the underlying CT (Fig 2A). Co-localization of RFP signals and NTPDaseII immunoproducts was evident in taste buds. However, ubiquitous NTPDaseII immunoreactivity in the taste buds made it difficult to distinguish the RFP^+^NTPDaseII^+^ cells from the RFP^+^NTPDaseII^−^ cells. The type II (α-Gustducin^+^) (Fig 2B) and type III (SNAP25^+^) (Fig 2C) cells were distinctly labeled and easily identifiable so these cells were used for further quantification of cell profiles.

Proportions of RFP^+^ specific taste cell types in fungiform, foliate and circumvallate papillae, relative to total *P0-Cre* labeled RFP^+^ cell profiles (column A) or relative to the type II or III cells (column B) are shown in Table 1. The percentages were calculated based on the number of all the counted cells from different mice. The percentage of labeled RFP^+^NTPDaseII^+^ relative to RFP^+^ taste bud cells was an extrapolation from quantification analysis of type II and III cell labeling.

**Table 1:** Proportion of *P0-Cre/RFP* labeled specific type (I, II, III)of taste bud cell profiles.

P0-Cre/RFP labeled taste bud cells	A: % (vs. n = number of RFP+ taste bud cells)			B: % (vs. n = number of specific type taste cells)	
	(counted separately for II and IIIfrom different sets of sections)			(α-Gustducin+ or SNAP25+)	
	I (extrapolation)	II (n)	III (n)	II (n)	III (n)
**Fungiform**	60	30 (183)	10 (423)	21 (255)	48 (88)
**Foliate**	64	7 (365)	29 (918)	8 (333)	63 (431)
**Circumvallate**	58	12 (479)	30 (1039)	10 (541)	33 (954)
**Overall in lingual taste buds**	**61**	**13 (1027)**	**26 (2380)**	**12 (1129)**	**42 (1473)**

Variability was observed in the three types of taste papillae. Of all RFP^+^ cell profiles in lingual taste buds, 61% were type I (extrapolation), 13% type II and 26% type III cells (Table 1, column A). Relative to type II or III cell profiles (Table 1, column B), the percentages of double-labeled cells were also obtained. The type III cells exhibited a high percentage of RFP^+^ cell profiles. In overall quantified lingual taste buds, 42% of type III cells were labeled with RFP signals, in contrast to a 12% of type II RFP^+^ cells.

### *Dermo1-Cre* also labeled mature taste bud cells and underlying connective tissue in the tongue and soft palate

The abundant distribution of *P0-Cre/RFP* labeled cells in taste buds and underlying CT in the absence of labeled surrounding epithelium strongly suggests a novel perspective regarding taste bud cell origin, i.e., a population of taste bud cells share the same origin as the underlying mesenchymal CT. To confirm this significant finding, we used another independent mouse model, *Dermo1-Cre* [27], in which *Cre* is driven by the endogenous promoter of *Dermo1* that is expressed in the mesenchyme of embryonic tongue [28].

**Fig 3:**
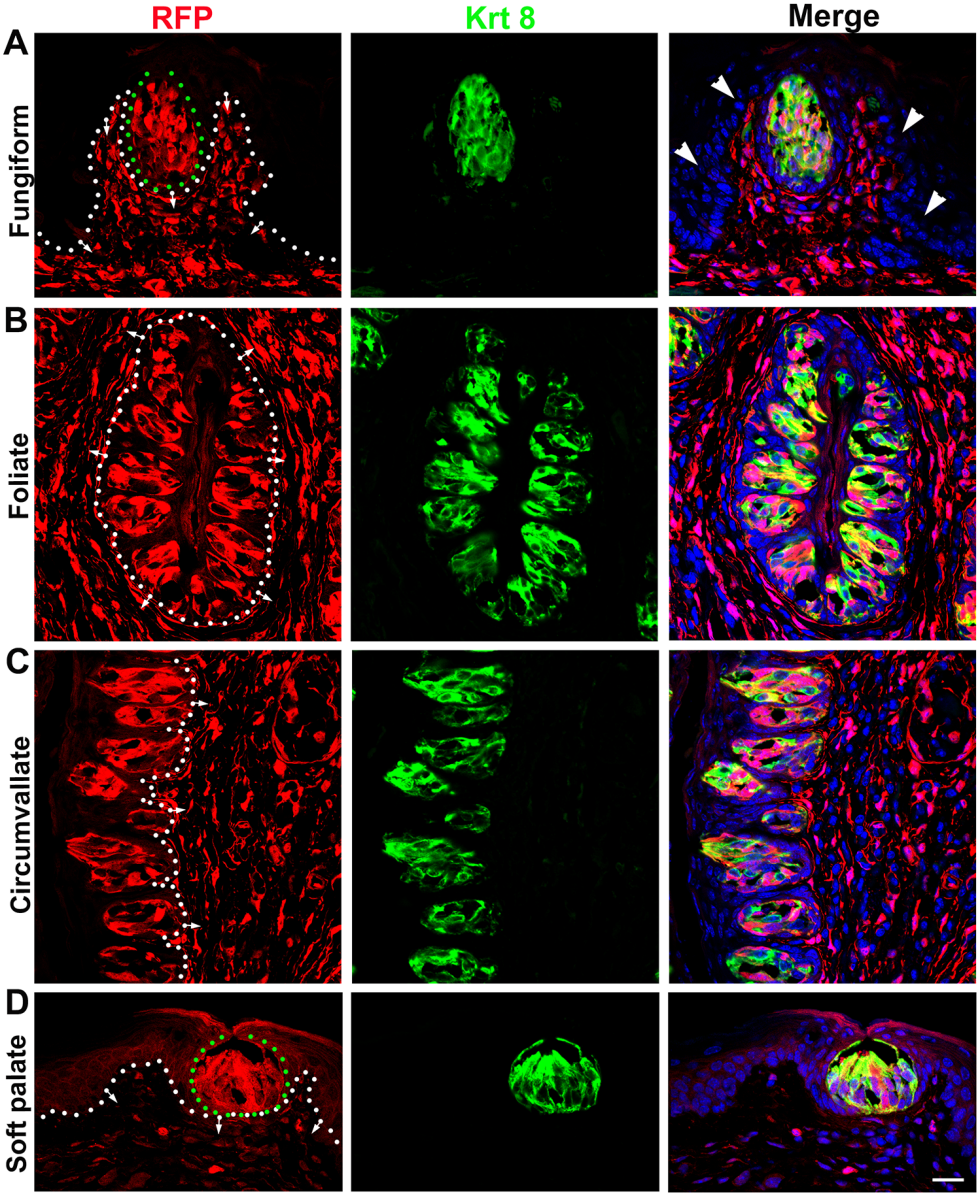
*Dermo1-Cre* labeled abundant population of taste bud cells. In adult *Dermo1-Cre/RFP* mice, RFP^+^ cells were abundantly distributed in mature taste buds labeled by Krt8 (green) and extensive in the underlying connective tissue of lingual fungiform (A), foliate (B), circumvallate (C) papillae and soft palate (D). Sections were counterstained with DAPI (blue). White dotted lines demarcate the epithelium from underlying connective tissue with arrows pointing to the connective tissue. Green dotted circles outline Fungiform and Soft palate taste buds. Arrowheads in A point to the unlabeled RFP^−^ epithelial cells outside of the taste bud. Scale bar: 20 μm for all images (single plane laser-scanning confocal).

**Fig 4:**
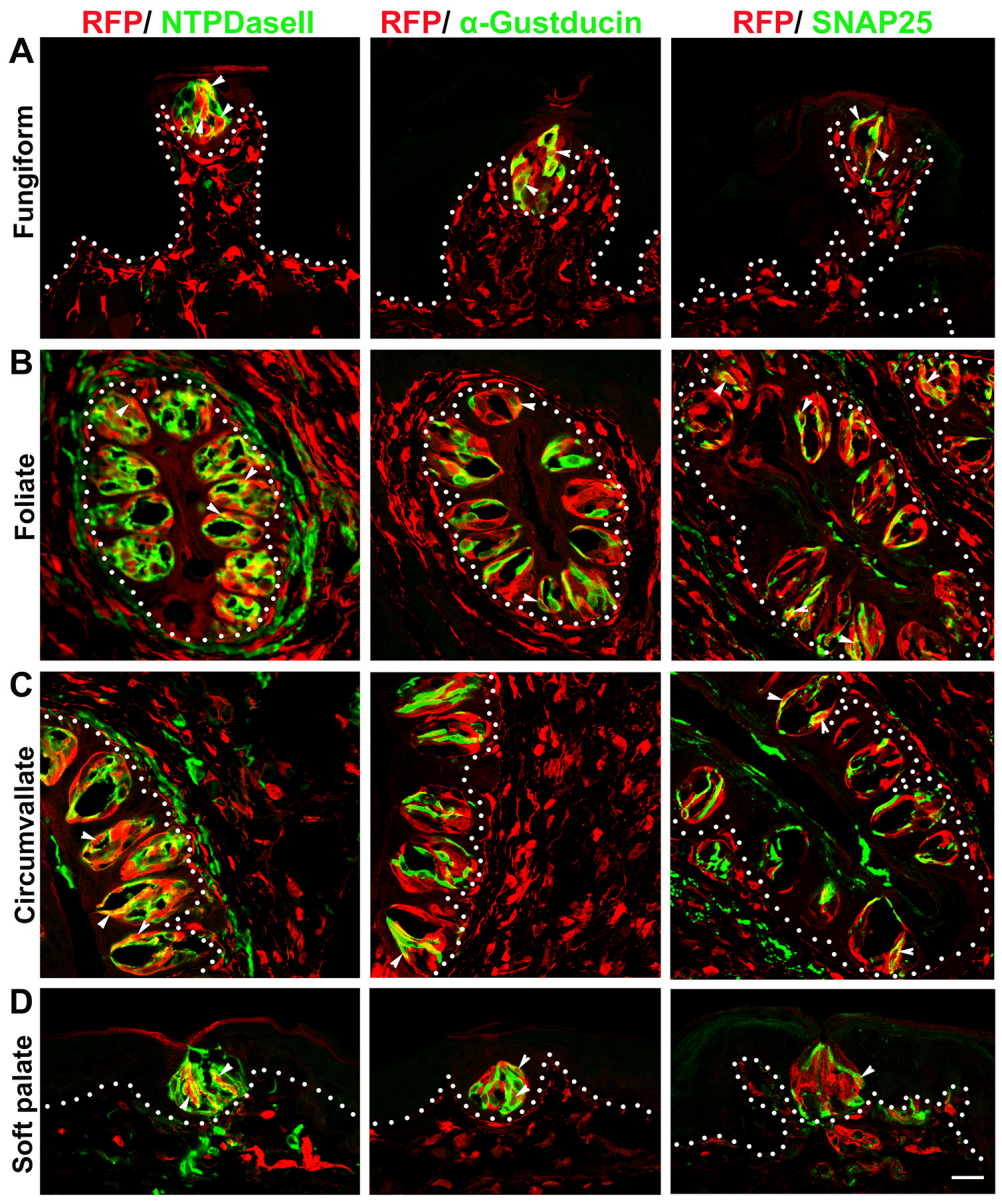
*Dermo1-Cre* labeled all three types (I, II, III) of taste bud cells in adult mice. RFP^+^ signals were co-localized with markers for specific taste cell types (white arrowheads), i.e., NTPDaseII for type I cells, α-Gustducin for type II cells and SNAP25 for type III cells in the lingual (A, B, C) and palatal (D) taste buds. White dotted lines demarcate the epithelium from underlying connective tissue. Scale bar: 20 μm for all images (single plane laser-scanning confocal).

Our data from the *Dermo1-Cre/RFP* model were consistent with our observations from *P0-Cre/RFP* mice for: (1) the abundant distribution of labeled taste bud cells and underlying CT (Fig 3) in the absence of labeled surrounding epithelial cells (Fig 3A, arrowheads); and (2) the co-localization of RFP signals with markers for differentiated type I, II, III taste bud cells (Fig 4, arrowheads).

In brief, a large population of *Dermo1-Cre* labeled RFP^+^ cells was seen in mature taste buds and co-localized with the pan taste cell marker Krt8 in lingual taste papillae, i.e., in fungiform, foliate and circumvallate, and soft palate (Fig 3). Of note, the epithelial cells that surround taste buds were not labeled (Fig 3A, arrowheads). Instead, bright RFP^+^ cells were widely distributed in the underlying CT, and were especially dense in the lamina propria and CT core of taste papillae. Moreover, RFP labels were co-localized with markers for all three differentiated taste cell types (Fig 4, arrowheads), i.e., NTPDaseII for type I, α-Gustducin for type II, and SNAP25 for type III.

### *Vimentin-CreER* labeled cells were distributed within taste buds after tamoxifen administration

To demonstrate that the underlying CT cells continuously contribute to the renewal of mature taste buds, we used an inducible *Cre* mouse model, *Vimentin-CreER* in which *Cre* activity was driven by the endogenous promoter of *Vimentin* that is expressed in the tongue CT.

**Fig 5:**
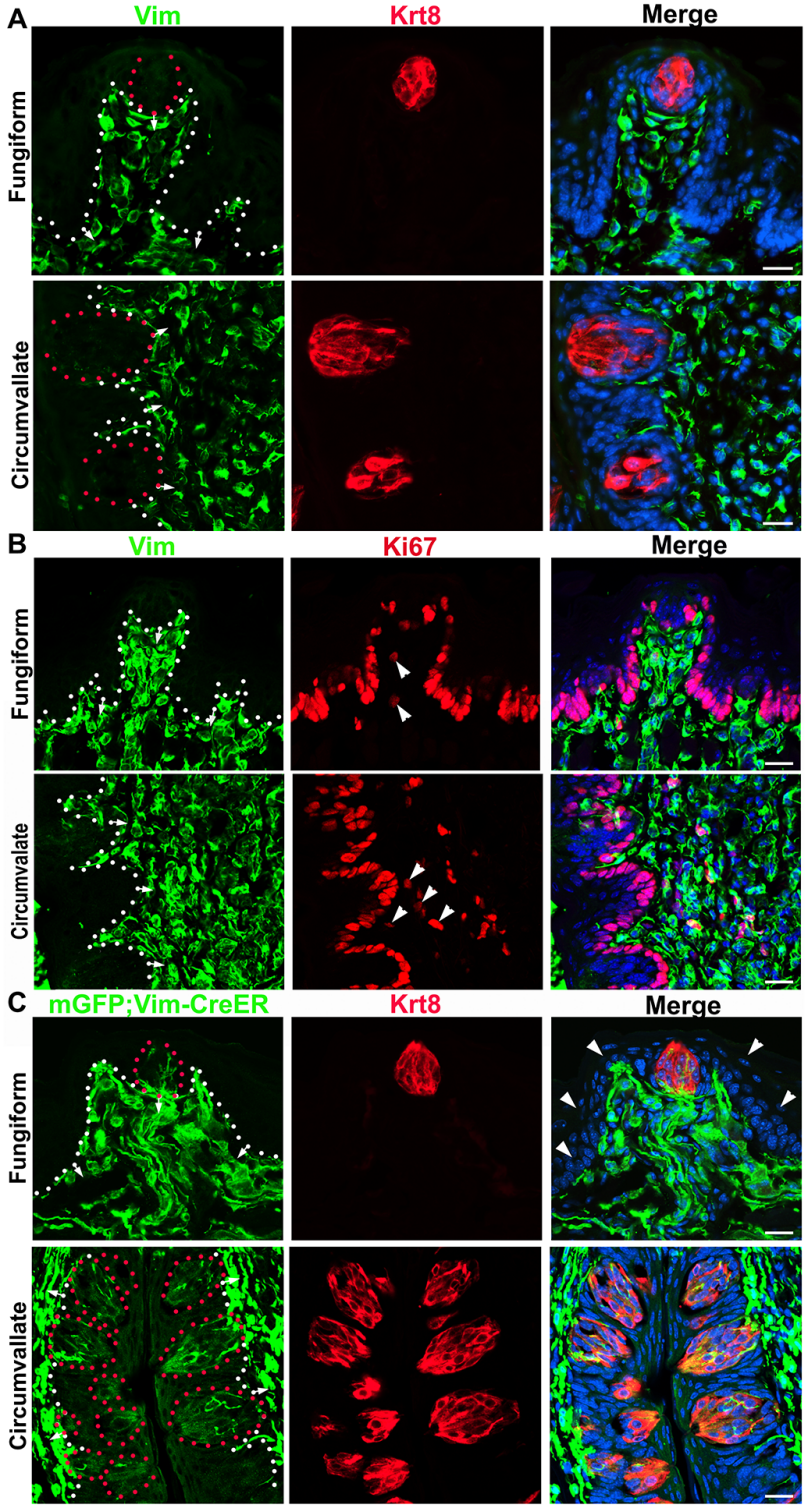
Derivation of taste bud cells from *Vimentin*-expressing cells in the underlying connective tissue. **A, B**: In adult wild type mice, the immunoreactivity of Vimentin (Vim, green) was extensively distributed in the tongue connective tissue but not in the epithelium or Krt8^+^ taste bud cells (red in A). A subpopulation of Vimentin^+^ cells were also labeled with proliferating cell marker Ki67 (red in B, arrowheads). **C**: In adult *Vimentin-CreER/mGFP* mouse, tamoxifen induced mGFP^+^ cells were seen within the Krt8 (red) labeled taste buds in fungiform and circumvallate papillae in addition to the extensive distribution of mGFP^+^ cells in the underlying connective tissue. White dots demarcate the border between epithelium and lamina propria with arrows pointing to the connective tissues. Red dotted lines in A and C encircle the taste buds. Arrowheads in C (Fungiform) point to the unlabeled mGFP^−^ epithelial cells. Scale bars: 20 μm for all images (fluorescent light microphotographs in A and single plane confocal images in B and C).

In adult wild type mice, Vimentin immunoreactivity was widely seen in the underlying CT of the tongue but not in taste buds nor in the surrounding epithelium (Fig 5A). Double immunolabeled Vimentin^+^Ki67^+^ cells were observed in a small population of connective tissue cells adjacently below the taste buds (Fig 5B, arrowheads). However, in the adult (3-month) *Vimentin-CreER/mGFP* mice, mGFP^+^ cells were apparently distributed within lingual taste buds in fungiform and circumvallate (Fig 5C) papillae in addition to the underlying CT cells at 12 days after the first tamoxifen treatment. In the foliate taste buds, mGFP^+^ cells were not as clear as in fungiform and circumvallate (data not shown). Again, the epithelial cells surrounding the taste buds were not labeled (Fig 5C, Fungiform, arrowheads).

## Discussion

### A population of taste bud cells originate from underlying connective tissue in tongue and soft palate

Mammalian taste bud cells, specialized gustatory sensory organs that primarily reside in the epithelium of lingual taste papillae and soft palate [32], have both epithelial and neuronal features. It has been widely accepted that taste bud cells are derived from the local surrounding epithelium [3–7, 33], which is distinctive among most, if not all, other sensory organs that have a neural origin including neural crest (NC). Here, we report a previously unrecognized origin of taste bud cells, i.e., the adjacent underlying connective tissue (CT), potentially of NC origin based on multiple lines of evidence from the present study: (1) concurrent distribution of labeled cells in taste buds and underlying CT in *P0-Cre/RFP* and *Dermo1-Cre/RFP* mice; (2) distribution of labeled taste bud cells in the absence of labeling in the surrounding epithelium; (3) distribution of Vimentin^+^ CT cell derived cells (*Vimentin-CreER/mGFP*) in the taste buds. Furthermore, distribution of *Vimentin-CreER* labeled cells in taste buds in adult mice also confirmed the continuous contribution of CT cells to the renewal of mature taste buds. These novel findings bring new perspective and better understanding of the precursor sources for taste bud formation and renewal, i.e., from both the underlying CT and the surrounding epithelium.

The distribution of Vimentin immunoreactivity in the CT cells versus absence in taste buds in mouse tongue is similar to that in human but distinct from that in chicken, i.e., sustained Vimentin expression in both the taste bud cells and underlying CT [34]. Witt *et al*. [34] proposed that “the mechanisms of taste bud differentiation from source tissues may differ among vertebrates of different taxa”. However, our data support the idea that similarly to chicken, the mammalian CT cells contribute to taste buds, and that differently from chicken, the mammalian CT cells lose the Vimentin expression after their migration and differention to taste bud cells.

It is noteworthy that the absence of *P0-Cre* driven RFP labeling in the tongue epithelium outside of the taste buds is different from our observation in the previous report (Liu et al., 2012) using the *R26R/lacZ* reporter in embryos and young postnatal animals (P1-10), i.e., X-Gal stained clusters of epithelial cells in the interpapillary space. We believe that the single-plane confocal microscopy in the present study provides a more accurate analysis of the distribution of RFP^+^ signals driven by *P0-Cre*. Similar distributions were observed in the *Dermo1-Cre* and *Vimentin-CreER* mouse models.

### Derivation of lingual and palatal mesenchyme/connective tissue

The mammalian tongue and soft palate contain striated muscles that are compartmentalized by CT with traversing blood vessels and nerve fibers. It is well known that lingual and palatal striated muscle cells are derived from mesoderm [21, 35] and that the CT cells are largely derived from cranial NC [21, 36]. In both *P0-Cre/RFP* and *Dermo1-Cre/RFP* mouse lines, the mesoderm derived muscles were not labeled indicating that the labeled CT and taste bud cells are most likely from NC.

Important contributions of NC cells have been demonstrated in the formation of mammalian craniofacial structures, including in the branchial arches [37, 38] where the tongue forms. Consistently in the previous reports [23, 37] and present study, labeled cells were extensively distributed in the tongue mesenchyme/CT across a broad range of stages (from early embryos to mature adult mice) using multiple reporters (*lacZ*, *GFP*, *R26-tdTomato*) driven by *Cre* activity under the control of different promoters (*Wnt1-Cre*, *P0-Cre*, *Dermo1-Cre*) to map NC derivatives.

Cranial NC plays important roles in the tongue formation. It has been shown that the cranial NC derived cells closely interact with myogenic progenitors in tongue myogenesis and morphogenesis through multiple signaling pathways, e.g., Dlx, TGFb, FGF [21]. In the present study, we demonstrated a novel role of mesenchymal/CT cells that are primarily from cranial NC, i.e., migration and differentiation to taste bud cells. CT are comprised of multiple cell types that include fibroblasts, intrinsic ganglion neurons, blood vessels, and Schwann cells that myelinate nerve fibers. Schwann cells, derived from NC, are important for the development and myelination of peripheral nerves. It has been reported recently that tissue injury can lead to the dedifferentiation of Schwann cells and then differentiation to neurons [39, 40]. Further studies are needed to characterize the specific cell types in the CT that contribute to taste buds.

Intriguingly, *Wnt1-Cre* labeled cells are extensive in the tongue mesenchyme but rare in the early taste buds (17). It is too early to provide a reasonable speculation for the profound differences between *P0-Cre* and *Dermo1-Cre* versus *Wnt1-Cre* in labeling taste bud cells, i.e., abundant in both *P0-Cre* and *Dermo1-Cre* versus rare in *Wnt1-Cre* [23]. Inconsistencies in labeling NC derivatives in other organs with different *Cre* driver models have also been reported presumably because of the variation in labeled NC cell populations [41–43]. Indeed, the established mouse lines for mapping NC derivatives do not label all NC derived cells, or label NC derived cells exclusively from other cell lineages. *Wnt1-Cre*, widely used and taken as a “gold standard” for labeling NC derivatives, has been found recently to cause ectopic activation of Wnt signaling and defects of midbrain development [44]. It is not clear whether the alterations caused by the *Wnt1-Cre* transgene lead to the absence of taste bud labeling. A detailed examination of cell types that are labeled by *Wnt1-Cre* and newly developed *Wnt1-Cre2* [44] versus *P0-Cre* and *Dermo1-Cre* may lead to a better understanding of which cell type(s) in the underlying CT contribute to taste buds.

### Proportion of taste bud cells derived from underlying connective tissue

Taste buds are primarily located in the tongue and soft palate within the oral cavity in mice [32]. A significant contribution of CT to taste buds in both tongue and soft palate is supported by the abundant distribution of *P0-Cre* and *Dermo1-Cre* labeled cells in taste buds along with the underlying CT in all three types of lingual taste papillae and in soft palate. By tracking individual fungiform taste buds, our quantitative analysis showed that the majority of fungiform taste buds, 88% in adult and 96% in young mice, are comprised of labeled cells that share the same origin of underlying CT. The average proportions of RFP^+^ taste bud cell profiles ranged from 24–39%. Our data suggest that a significant population of taste bud cells are derived from the underlying CT that is of NC origin.

Of note, *P0-Cre* and *Dermo1-Cre* labeled cells were sustained in mature taste buds at 16 weeks, indicating the existence of stem/progenitor cells in the CT for taste bud renewal and maintenance. This idea is supported by our observation that a small proportion of Vimentin^+^ cells in the CT core of taste papillae are also positive for the proliferating marker Ki67. Indeed, labeling of taste bud cells with *Vimentin-CreER* in adult mice supports the hypothesis that underlying CT cells contribute to the continuous turnover of taste bud cells that have an average half-life of 8–12 days [22]. Mii *et al* [45] recently reported the distribution of nestin-expressing multipotent stem cells in the CT core of fungiform papillae. These cells co-express the NC cell marker p75 and are immediately below the taste buds. Combined with our data, we propose that NC derived stem cells exist in the underlying CT and contribute to the renewal of taste buds.

### Types of taste bud cells derived from underlying connective tissue

Taste bud cells are heterogeneous structurally (types I, II, III, IV) [8–10] and functionally (epithelial-, neuronal-, and glial-like) [11–14] which suggests distinct lineages of taste bud cells [15]. It is well known that type I (glial-like) cells are the most abundant and serve as supporting cells; whereas type II cells are less abundant and responsible for transducing sweet, bitter and umami taste stimuli through a non-traditional contact with the sensory nerve endings. Type III (neuronal-like) cells are the least abundant cell type and important for sour taste [31, 46].

Specific markers for the three types of differentiated taste bud cells are useful in defining the contribution of underlying CT to specific taste bud cell type(s), e.g., NTPDaseII for type I [47], α-Gustducin for type II [48, 49], SNAP25 for type III [50]. The observation that markers for every differentiated taste cell type were co-localized with RFP signals in both *P0-Cre/RFP* and *Dermo1-Cre/RFP* mouse lines demonstrates that the underlying CT contributes to all types of differentiated taste bud cells, i.e., type I, II and III in all three types of lingual taste papillae and soft palate.

Our quantitative data for the composition of overall *P0-Cre/RFP*^+^ cells, i.e., 61% type I (extrapolation), 13% type II, and 26% type III, indicate an uneven differentiation of taste cell types from taste bud progenitors in the underlying CT, a distribution that favors neuronal-like type III cells. Indeed, a higher percentage (42%) of type III cell profiles in lingual taste buds were labeled with *P0-Cre/RFP* versus 12% RFP^+^ type II. This supports the idea that the taste bud progenitors in the underlying CT have a neural origin, more likely NC, and tend to differentiate toward neuronal cells, e.g., type III taste bud cells.

### Dual origin of taste bud cells from both surrounding epithelium and underlying connective tissue

In light of previous reports [5, 6, 18, 19, 23, 34, 51] and our present study, we propose a dual origin of taste bud cells from both the surrounding epithelium, i.e., K14^+^K5^+^Trp63^+^Sox2^+^(low)Lgr5^+^ basal cells, and the underlying CT, i.e., Vimentin^+^ cells that are most likely derived from NC. Although quantitative data for contributions of *K14-CreER* labeled cells to taste buds were not provided in the report, different distribution patterns/types of taste bud labeling were observed, i.e., fully, partially, or absent *K14-CreER* labeling (6). Combined with our fate mapping analysis in the present study, the data support a compatible distribution of taste bud cells from both origins, i.e., the majority of taste buds have a mixed population with both origins, and a small population of taste buds are primarily from either surrounding epithelium or underlying CT.

Significantly, our data using three transgenic mouse lines bring forward a novel progenitor source of taste bud cells. It is important to understand how this population of CT cells can be regulated to migrate and differentiate to taste bud cells, and how these cells interact with the surrounding epithelium for the proper formation and renewal of taste buds. Our finding that the underlying CT contributes to taste bud cells provides a new insight into taste bud formation and renewal.

## Supporting Information

S1 Fig Representative photomicrographs taken with a fluorescent light microscope illustrates the absence of RFP^+^ cells in all the *P0-Cre(-)/RFP(+)* mouse tissues examined, i.e., fungiform, foliate, circumvallate and soft palate. Taste bud cells were labeled with Krt8 immunoreactivity (green) and sections were counterstained with DAPI (blue). Scale bar: 50 μm for all images.
(TIF)

S2 Fig Distribution of *P0-Cre/RFP* labeled RFP^+^ cells in a fungiform papilla (A) and the soft palate (B). Taste bud cells (encircled by green dots) were labeled with Krt8 immunoreactivity (green) and sections were counterstained with DAPI (blue). White dots demarcate the epithelium from connective tissue pointed by the short arrows. White arrowheads point to the unlabeled epithelium outside of taste buds, i.e., in between-papilla lingual or between-bud palatal epithelium. RFP signals were not observed in striated muscle cells (long arrows). Scale bar: 40 μm for all images (single plane laser-scanning confocal).
(TIF)

S3 Fig Distribution of *P0-Cre/RFP* labeled RFP^+^Krt8^+^ taste bud cells in fungiform taste buds and underlying connective tissue at week 2 (A), 4 (B) and 16 (C). White dots demarcate the epithelium from connective tissue. Short arrows point to the underlying connective tissue. Green dots encircle the taste buds. White arrowheads point to the unlabeled epithelium outside of taste buds in the fungiform papillae. Scale bar: 20 μm for all images.
(TIF)
