# Supplementary figures and images for: Contribution of Underlying Connective Tissue Cells to Taste Buds in Mouse Tongue and Soft Palate
Source: PLoS One. 2016 Jan 7;11(1):e0146475. doi: 10.1371/journal.pone.0146475 (PMC4704779; doi:10.1371/journal.pone.0146475)

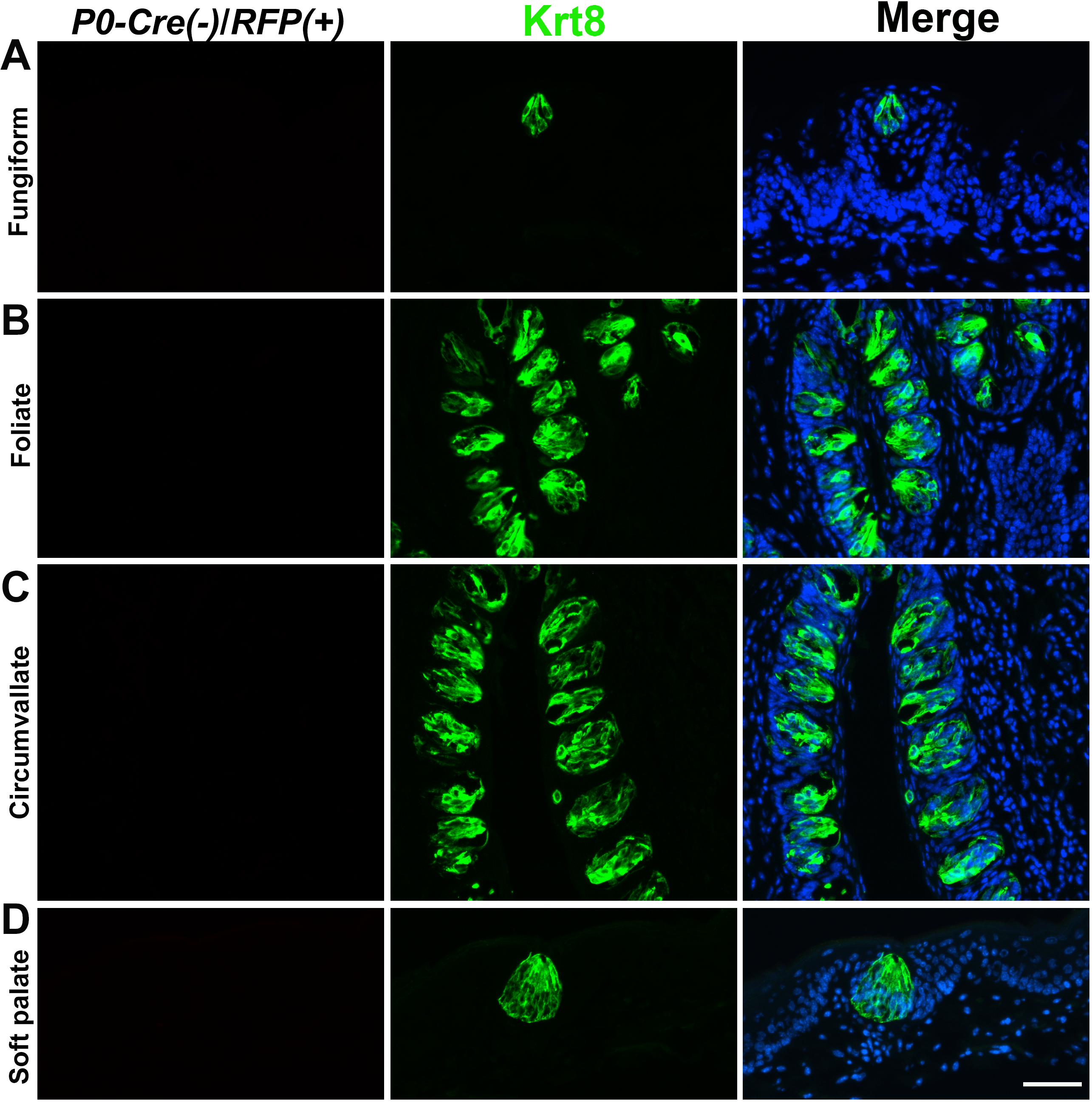

Supplement: S1 Fig — Taste bud cells were labeled with Krt8 immunoreactivity (green) and sections were counterstained with DAPI (blue). Scale bar: 50 μm for all images. (TIF) [file pone.0146475.s001.tif]

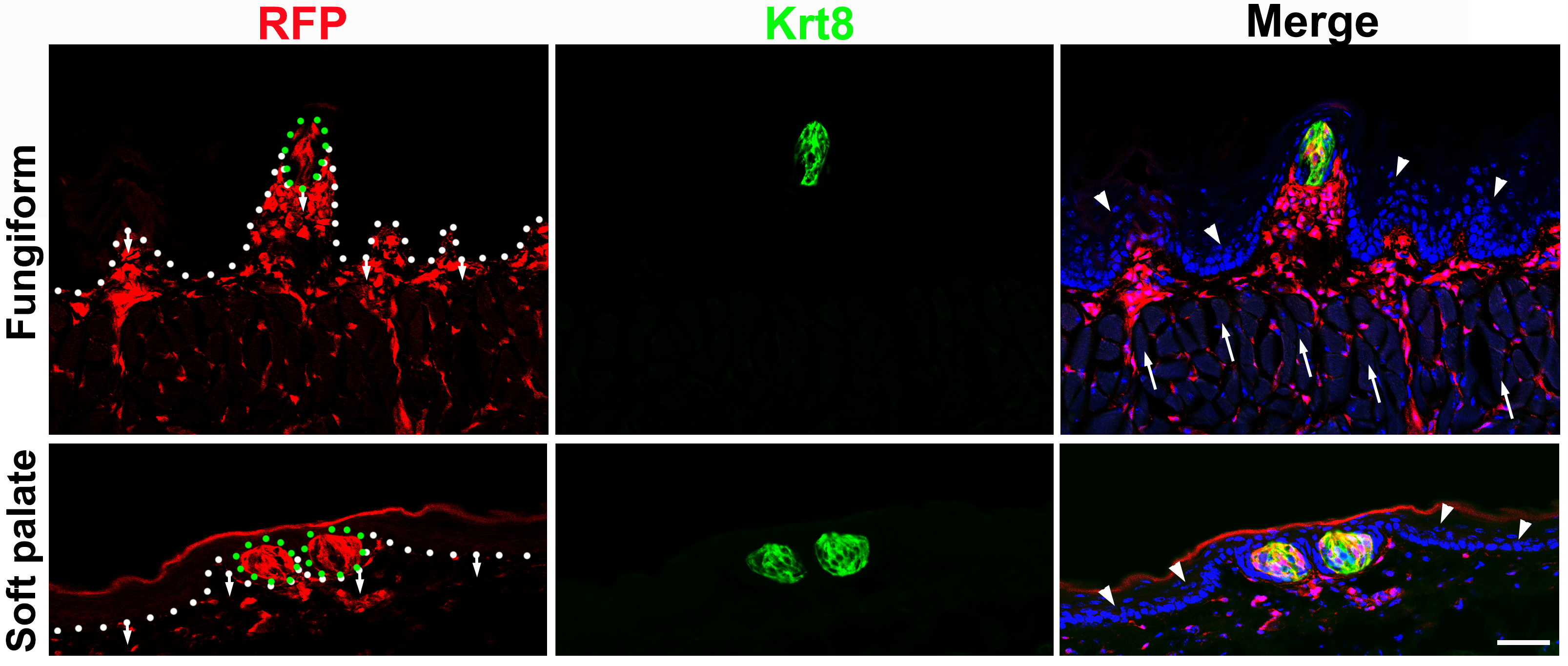

Supplement: S2 Fig — Taste bud cells (encircled by green dots) were labeled with Krt8 immunoreactivity (green) and sections were counterstained with DAPI (blue). White dots demarcate the epithelium from connective tissue pointed by the short arrows. White arrowheads point to the unlabeled epithelium outside of taste buds, i.e., in between-papilla lingual or between-bud palatal epithelium. RFP signals were not observed in striated muscle cells (long arrows). Scale bar: 40 μm for all images (single plane laser-scanning confocal). (TIF) [file pone.0146475.s002.tif]

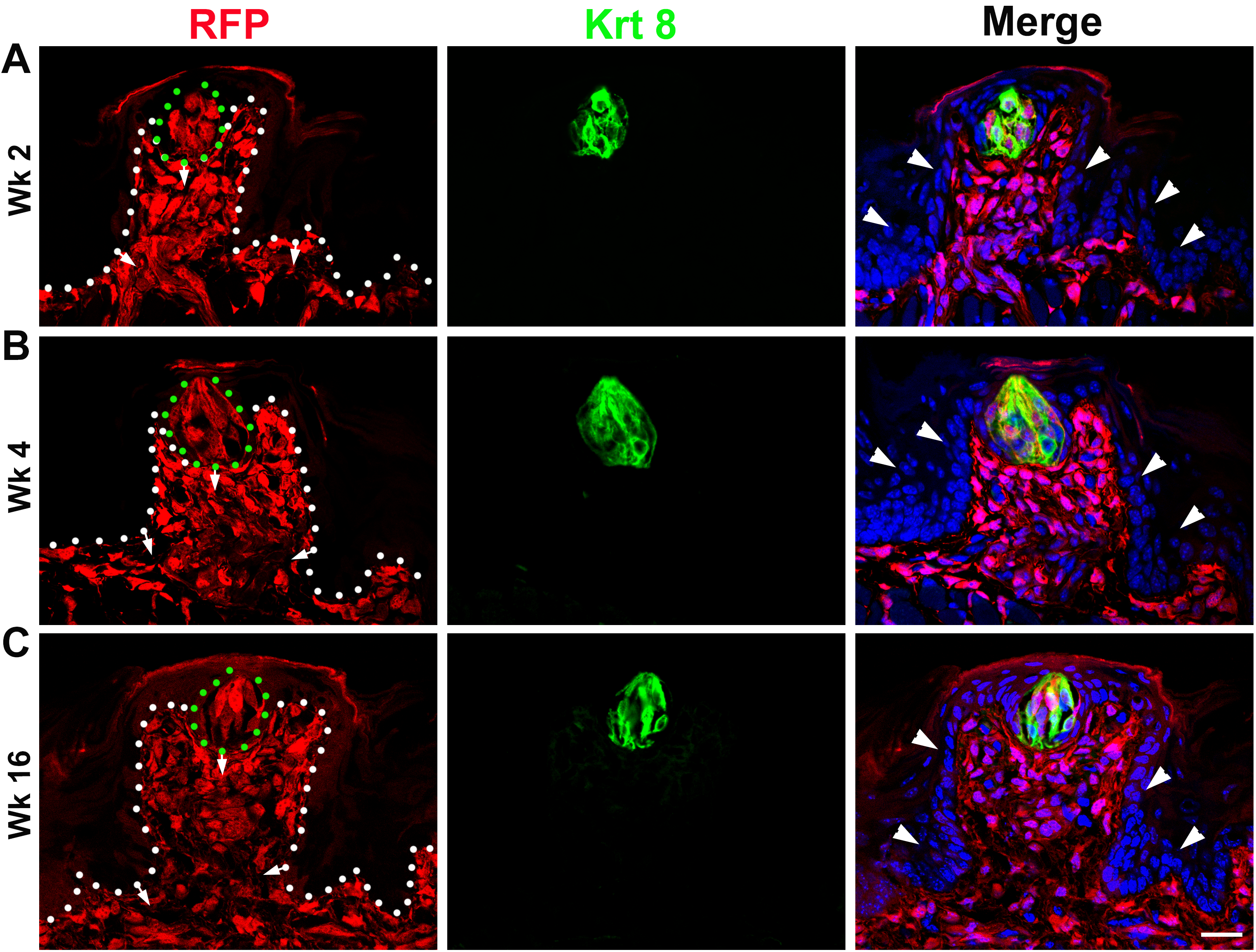

Supplement: S3 Fig — White dots demarcate the epithelium from connective tissue. Short arrows point to the underlying connective tissue. Green dots encircle the taste buds. White arrowheads point to the unlabeled epithelium outside of taste buds in the fungiform papillae. Scale bar: 20 μm for all images. (TIF) [file pone.0146475.s003.tif]
